# Evaluation Frequency of Human Papillomavirus and Its Related Genotypes in Women of the General Population Living in 11 Provinces of Iran

**DOI:** 10.1155/2022/8668557

**Published:** 2022-04-26

**Authors:** Somayeh Hassani, Proshat Sadat Nadji, Anita Mohseni, Marjan Rahnamaye Farzami, Siamak Mirab Samiee, Makan Sadr, Seyed Alireza Nadji

**Affiliations:** ^1^Virology Research Center, National Research Institute of Tuberculosis and Lung Diseases (NRITLD), Shahid Beheshti University of Medical Sciences, Tehran, Iran; ^2^Department of Microbiology, Faculty of Pharmacy and Pharmaceutical Sciences, Tehran Medical Sciences, Islamic Azad University, Tehran, Iran; ^3^Reference Health Laboratory Research Center, Ministry of Health and Medical Education, Tehran, Iran

## Abstract

**Introduction:**

Persistent infection with one of the most high-risk genotypes of human papillomavirus causes all cases of cervical cancer and a significant proportion of other genital cancers. The HPV virus, unlike any other infection that leads to cancer, is transmitted only through sexual intercourse and is less affected by the general changes and development in lifestyle and medical standards, so only vaccination and screening can prevent the HPV virus and cancers caused by it. Therefore, determining the prevalence and distribution of HPV genotypes are of utmost importance in screening strategies regarding cervical cancer and vaccination decisions against HPV that vary based on the geographical and cultural characteristics of the study area. As a result, this study aimed to determine the frequency of human papillomavirus and the distribution of this virus's genotypes in the general population of women living in 11 provinces of Iran.

**Materials and Methods:**

This study is a community-based survey study. Sampling was done by the cluster sampling method. Women aged 15–59 years old from the general population living in 11 provinces of Iran were included in the study after considering the inclusion and exclusion criteria. Data were collected using a questionnaire and vaginal examination. The study was performed on 2562 vaginal specimens that were referred to the laboratory of the present study. HPV genome was detected by the nested MY-GP method and papillomavirus genotyping was performed using the PCR multiplex method to identify 19 papillomavirus genotypes.

**Results:**

The general prevalence of HPV in the 11 provinces was obtained at 2.4% (108 out of 2562 people). The highest prevalence of the virus was in the age group of 25–34 years. The prevalence of HPV was statistically significant among different provinces. Hormozgan province with 22 cases (5.9%) had the highest and Isfahan province with 6 cases (2.2%) had the lowest incidence of HPV. The prevalence of high-risk HPV and medium-risk HPV is 3%, and the prevalence of low-risk HPV was estimated to be 2.1% of the total female population. Also, the highest prevalence was related to genotype 16.

**Conclusion:**

According to the high prevalence of the HPV virus in young age groups in Iran, it is necessary to pay attention to screening programs to reduce the incidence of cervical cancer.

## 1. Introduction

Human papillomavirus (HPV) is a small virus with a double-stranded DNA genome that tends to attach to the squamous epithelium of the skin layers [1] and mucous membranes of the upper gastrointestinal tract, respiratory tract, and genital tract that causing genital warts, laryngeal papillomas, and certain types of cancer [2]. HPV is responsible for approximately 5.2% of all human cancers (including cancers of the genitals, anus, and oropharynx) worldwide [3]. Getting infected by the HPV virus is one of the most common sexually transmitted infections (STD) in women and men [4–6] and many people get the virus at some point in their lives [7]. The infection rate of HPV and the cancers as a result of it are more common in women than men [8]. The risk of contracting the virus during a woman's lifetime is about 80% [9]. But the chance of getting cancer depends on if the virus has the potential to cause it [2, 9]. Based on the cancer-causing abilities of the HPV virus, virus-related genotypes are divided into two groups: high-risk and low-risk groups [2]. Persistent infections with high-risk virus genotypes (16, 18, 31, 33, 35, 39, 45, 51, 52, 56, 58, 59, 68) are the main etiological factor in the development of cervical cancer [4] and a significant proportion of other genital cancers and head and neck cancers in men and women [10].

The rate of incidence and prevalence of cancers associated with the HPV virus is on the rise [2]. In 2018, cervical cancer had 570,000 cases and 311,000 deaths, ranking it the fourth in terms of incidence and mortality among all types of gynecological cancers worldwide [11]. The prevalence of cancer in developing countries is due to a lack of proper screening programs [12]. Also, more than 85% of the global burden of cervical cancer is in developing countries [9]. Although in Iran cervical cancer is not one of the top 10 most common cancers among women, according to estimates made in 2012, 947 new cases of cervical cancer are diagnosed year by year in this country, which is a significant number [13]. Persistent infection alongside the HPV virus is the essential factor in causing cervical cancer [8]; therefore, determining the prevalence and distribution of genotypes of this virus, through planning and policy for screening and vaccination, is necessary to prevent cervical cancer. Because the HPV virus, unlike any other infection that leads to cancer (such as *Helicobacter pylori*, hepatitis B, C), is transmitted only through sexual intercourse and is less affected by the general changes and development in lifestyle and medical standards, so only vaccination and screening can prevent the HPV virus and cancers caused by it [14]. In this regard, many studies have been conducted around the world. They have shown that the prevalence of the HPV virus [9] and the prevalence and distribution of high-risk genotypes [15] of this virus vary based on the geographical and cultural characteristics of the study area. The analysis of the prevalence of the virus worldwide also shows a difference of 20 times in the prevalence and distribution of the virus in different geographical regions in the four continents of Asia, Africa, Europe, and South America [15]. Therefore, to achieve preventive goals, it is necessary to study the prevalence and distribution of the virus separately in different countries as well as in different regions of a country. Keeping in mind the said information, and considering that few studies have been conducted in Iran regarding this field, the present study was conducted to determine the frequency of human papillomavirus and the distribution of genotypes of this virus in the general population of women living in 11 provinces of Iran.

## 2. Materials and Methods

### 2.1. Study Design, Sample Size, and Sample Collection

 The present study is a community-based survey study. The aim was to determine the frequency of human papillomavirus in Iranian women and to know the distribution of genotypes of this virus in the general population of women living in different provinces of Iran. 2631 samples were transferred to the laboratory for the present study. But the laboratory studies were performed on 2562 samples that had the necessary quality for analysis. Cluster sampling was done by first randomly selecting 11 universities from among the country's medical universities; then in each university, 3 urban areas and 2 rural areas that had the maximum difference in economic and social conditions were selected. Women who were members of households covered by selected medical university centers (Yazd, Ardabil, Gilan, Isfahan, Tabriz, Hormozgan, Semnan, Kerman, Khuzestan, Qazvin, and Kurdistan) were invited to participate in the HPV virus screening project as a public call for the prevention and early treatment of cervical cancer. In each cluster, the wide spectrum of the age group of the population was considered. Iranian females living in the mentioned geographical areas, ages 15–59 years, who were married or had a history of marriage, were precipitated in the study after signing a written consent. To gather data, a questionnaire, related to basic information, demographic characteristics, medical history, and lifestyle of the participants, as well as a section dedicated to clinical information related to gynecologic examination, was designed. The questionnaires were completed by the interviewers with a midwifery degree who had received the necessary training to conduct the interview. Vaginal examination and collecting vaginal discharge samples were performed by a gynecologist and a midwife in one of the selected centers' affiliate medical universities. Abnormal cases (cervicitis, abnormal discharge, pain, etc.) were recorded in a questionnaire, and the patients in question were further evaluated and treated. During speculum examination, sampling was performed using a cytobrush; then, the brush containing cervical cells was placed inside the thin prep vial with a volume of 20 ml and then was subjected to a Pap smear test and HPV screening. After recording the patient's profile on the vial and slide, the samples were placed in a special refrigerator (with a temperature of 2–4°C) to maintain the cold chain and were transferred to two designated laboratories for the HPV test. Among the collected samples, the sample of 2561 women participating in the study was transferred to the virology laboratory of Dr. Masih Daneshvari Hospital. The present study is the result of a statistical analysis of samples sent from 11 provinces (Ardabil, East Azerbaijan, Gilan, Hormozgan, Isfahan, Kerman, Khuzestan, Kurdistan, Qazvin, Semnan, and Yazd) to this laboratory.

### 2.2. Diagnosing HPV and Determination of Virus Genotypes

HPV genome was detected by the nested MY-GP method [16]; then, the positive samples were subjected to genotype using the home brow PCR multiplex method to identify 19 papillomavirus genotypes described before [17]. The HPV genotyping method was approved in two external performance trials conducted by the World Papilloma Network affiliated with the World Health Organization.

### 2.3. Ethical issues

The present study was approved by the ethics committee of the Iran Ministry of Health and Shahid Beheshti University of Medical Sciences. All necessary permits for research implementation were obtained from the Vice-Chancellor of research and submitted to selected university centers. The objectives of the study were provided in writing to the management of medical centers. All subjects were free to participate in or leave the study, and written consent was obtained from those wishing to participate in the study. Participants in whom the HPV virus, genital diseases, epithelial cell abnormalities, and malignancies were diagnosed were referred to medical centers to complete diagnostic and treatment procedures. In all stages of the project, the principle of confidentiality of participants' information was maintained.

### 2.4. Statistical Analysis

To analyze data, SPSS version 22 was used. In all tests, a *p* value of less than 0.05 was considered significant. To analyze the qualitative data of the test, *χ*^2^, and if needed ‘‘Fisher's Exact Test” was used. Due to the abnormal age distribution, the “Mann–Whitney test” was used to compare the average age.

## 3. Results

**Figure 1:**
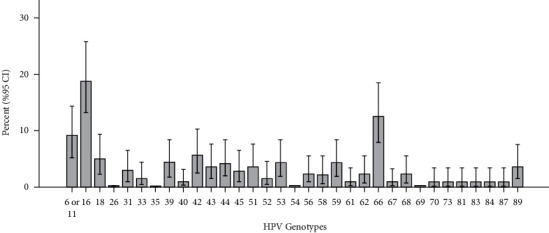
Prevalence of different HPV genotypes.

The study was performed on 2562 women aged 15–59 years from the general population living in 11 provinces of Iran after considering the inclusion and exclusion criteria. The general prevalence of HPV in 11 provinces was obtained at 4.2% (108 out of 2562 people). The prevalence of high-risk/intermediate-risk and low-risk HPV genotypes was estimated to be 3% and 1.2% of the total female population, respectively (Table 1). Among the HPV group infected by HPV, 83 people (76.9%) were infected a with single HPV genotype and 25 people (23.1%) had been infected with more of one of the virus genotypes (Table 2). The highest prevalence was related to genotype 16 (25%), followed by genotypes 66 (16.6%), 6/11 (12%), 42 (7.4%), 18 (6.4%), 39 (5.5%), 44 (5.5%), 53 (5.5%), 59 (5.5%), 43 (4.6%), 51 (4.6%), and 89 (4.6%) (Figure 1).

Table 1 shows characteristics, social status and fertility characteristics, and their relationship with the status of infections caused by HPV. The average age of women, in general, was 35/6 ± 10/9. There was no significant difference in the average age between the infected and noninfected groups (*p*=0.38). There was no statistically significant difference in the prevalence of HPV in different age groups (*p*=0.56). But the most prevalence of HPV was related to the age group of 25–34 years (4.5%), and the lowest prevalence was related to the age group of 55 years and above (3.4%). There was a significant difference between the prevalence of HPV in different provinces (*p*=0.33). Hormozgan province with 22 cases (9.5%) had the highest and Isfahan province with 6 cases (2.2%) showed the lowest prevalence of HPV. Based on previous studies, it was suggested that age, occupation, and education may affect the differences, in the prevalence of the virus among the provinces, so to examine the effect of these three variables, logistic regression analysis was performed. The results of regression showed that after adjustment based on the mentioned variables, the prevalence of the virus was significantly different between provinces, and the highest prevalence was still related to Hormozgan province (results are not shown). There was a significant difference in the prevalence of HPV between the two groups of employed and housewives (*p* < 0.05); HPV's prevalence was significantly higher in working women than in housewives. There was a significant difference in the prevalence of HPV based on the number of deliveries (*p* < 0.01), and the prevalence of HPV was lower in women who had more deliveries (the number of deliveries was 2 ± 2 in the group affected by HPV Vs. 3 ± 2 in the nonaffected group). Statistically speaking, there were no significant differences in the prevalence of HPV in educational status, marital status, history of abortion and stillbirth, and the history of genital infections.

**Figure 2:**
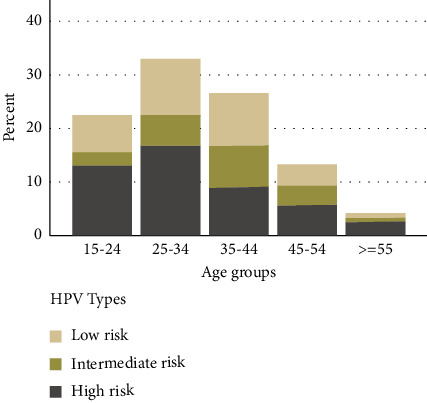
Prevalence of low-risk, medium-risk, and high-risk genotypes HPV based on age groups.

Figure 2 shows the prevalence of high-risk and low-risk HPV genotypes based on different age groups. As shown in the figure, the total prevalence of low-risk, medium-risk, and high-risk HPV genotypes in the age group of 25–34 years is more than in other age groups.

Table 2 shows the demographic characteristics, social status, and fertility characteristics in the HPV-affected group, based on high-risk, moderate, and low-risk genotypes. Among people with the HPV virus, the most observed genotypes were related to high-risk genotypes (50.9%), followed by low-risk genotypes (27.8%) and medium-risk genotypes (21.3%). There was no significant difference in the prevalence of different HPV genotypes between different age groups (*p*=0.53). Even though the prevalence of high-risk HPV genotypes was higher in Yazd and Semnan, no significant difference was seen in the prevalence of HPV genotypes among different provinces (*p*=0.33). Also, in terms of education, marriage, employment, history of abortion, stillbirth, and history of genital infections, no significant difference was observed between the three groups of high-risk, low-risk, and moderate-risk genotypes.

## 4. Discussions

Human papillomavirus plays an important role in the pathogenesis of cervical cancer, and awareness about the prevalence and distribution of its genotypes in adopting cervical cancer screening programs and deciding on vaccination against HPV is necessary. This study aimed to determine the frequency of human papillomavirus and the distribution of genotypes of the virus in the general population of women living in 11 provinces of Iran.

In the present study, the prevalence of HPV was obtained at 4.2%. Studies related to the prevalence of HPV in Iranian women have estimated the prevalence of the virus in different provinces to be 5.5%–36. 5% [18–24]. The virus has been reported to be more prevalent in women with sexually transmitted infections and cervical cancer, as well as in women with high-risk behaviors [25]. The result of a study in Rasht showed the prevalence of HPV virus in women with histopathological findings is 36.5% [23]. Results of a systematic review and meta-analysis have estimated the HPV outbreaks in Iran, in women with cervical infections (including *Mycoplasma genitalium*, *Chlamydia trachomatis*, and *Neisseria gonorrhea*) to be 38.6% [26]. Also, a study on Iranian female sex workers estimated the prevalence of the HPV virus to be at 49.1% [27]. The HPV prevalence in the present study was less than in other studies conducted in Iran. One of the reasons could be related to the population of women surveyed because the prevalence of the virus in the general population of women will be different from the prevalence of the virus in women referred to gynecological and sexually transmitted diseases clinics.

In connection with the prevalence of HPV in other countries, it should be borne in mind that the virus is associated with cervical cancer, so its prevalence in different countries is affected by the prevalence of cervical cancer [28]. In a meta-analysis, the global prevalence of HPV was shown to be 11.7%. The highest prevalence of HPV in this study is sub-Saharan Africa (24%), Eastern Europe (21%), and Latin America (16%), and the prevalence of HPV in West Asia was shown at 2% [14]. The low prevalence of cervical cancer and HPV virus infection in Muslim countries, including North Africa and West Asia, shows the importance of the impact of cultural environment and behavioral patterns on the incidence of this virus and cervical cancer [14]. In addition, it should be noted that the genetic diversity of HPV plays an important role in carcinogenic potential and HPV lineages and sublineages have different abilities to cause cervical cancer [29]. Iran is one of the nine countries with the lowest incidence and mortality from cervical cancer among the countries of West Asia and South-Central Asia (Gaza, Syria, Saudi Arabia, Yemen, Iraq, Lebanon, and Turkey also have low incidence and mortality of cervical cancer in the said area). It seems that, in addition to social issues and disapproval of extramarital affairs playing an important role in the lower prevalence of HPV and cervical cancer in countries in the Middle East [30], the genetic diversity of the virus is also influential. Mobini Kasheh et al. conducted a study to investigate the lineages of genotypes 16 and 18 of human papillomavirus in the samples obtained from the present study. The results of this study showed that the observed lineages and sublineages have less carcinogenic potential and that in itself can contribute to the lower prevalence of cervical cancer in Iranian women [31]. Keeping in mind the content in the paragraph above, the prevalence reported in the present study seems to be closer to reality.

In our study, the prevalence of high- and intermediate-risk HPV genotypes was 3% and the prevalence of low-risk HPV was estimated at 1.2%. Khodakarami et al. reported the prevalence of high-risk and low-risk HPV genotypes among the general population of women in Tehran and estimated to be 5.1% and 3.3%, respectively [19]. Jamdar et al. conducted a study on 2453 female Pap smears that were sent to the laboratory for screening. The overall prevalence of high-risk type HPV was estimated to be at 10.3% [32]. Differences in the overall prevalence of HPV, differences in sample size, and wider geographical area in the present study can affect the lower prevalence of virus genotypes than in other studies in Iran. In connection with the spread of high-risk strains of the virus in other countries, Rogovskaya et al. in a review study that included nine studies on women in the general population in Kazakhstan, Russia, and Uzbekistan showed that the prevalence of the high-risk HPV types varied from 11% to 40% among women in the general population [33]. The differences in the prevalence of cervical cancer between Iran and these countries are influenced by the differences in the prevalence of the high-risk type of HPV [28].

In the present study, the highest prevalence was related to genotype 16, followed by genotypes 66, 6/11, 42, 18, 39, 44, 53, 59, 43, 51, and 89. Some studies in Iran have identified several virus-related genotypes. Hamkar et al. in a study on women in different regions of Iran with normal cervical cytology showed that genotypes 16, 18, 66, and 11 followed by 6, 26, 31, 35, 44, 45, 51, 52, 53, 56, 67, 68, 73, 84, 89, and 90, respectively, are the genotypes observed in Iranian women [24]. A meta-analysis showed that genotypes 16, 18, 5, 2, 31, and 58 are the most common genotypes reported worldwide [14].

In the present study, the prevalence of genotypes 16 and 18 in the total study population was 1.1% and 0.28% and was almost similar to other studies conducted in Iran. Farahmand et al. showed the prevalence of 16 and 18 genotypes in women without cervical cancer at 1.8% and 1.2%, respectively [34]. Malari et al. showed the prevalence of genotypes 16 and 18 in healthy Iranian women to be at 2.03% and 1.7% [21]. In the study of Khodakarami, the prevalence of type 16 and type 18 was reported to be 1.2% and 0.2% in the general population of women in Tehran [19]. Jamdar et al. reported that the prevalence of HPV type 16 and type 18 was 1.8% and 0.4%, respectively [32].

The prevalence of HPV has a significant difference between provinces. Hormozgan province with 22 cases (9.5%) had the highest and Isfahan province with 6 cases (2.2%) had the lowest incidence of HPV (*p* < 0.01). Many studies have examined the prevalence of HPV in different provinces of Iran, but a similar study that compared HPV in the provinces of the present study was not observed.

In the present study, there was no significant difference in the prevalence of HPV in different age groups. But the most prevalence of HPV was related to age groups of 25–34 years (4.5%) and the lowest prevalence was related to age groups 55 years and above (3.4%). The prevalence of HPV varies according to age in different countries and populations. Results of a review study by Smith et al. showed that the prevalence curve of HPV depending on the age and the geographical area can be descending, flat, or U Shape (increasing prevalence in youth and the elderly) [35]. Jamdar et al. showed a reduction in the prevalence of HPV with increasing age. In their study, the highest prevalence was related to people under 25 years old (15.6%), and the lowest prevalence was related to people over 44 years old (4.5%) [32]. The prevalence of HPV in Pakistan has decreased with age, and the highest prevalence of high-risk and low-risk genotypes was observed in the age group of 35–45 years (41.67%), and the lowest prevalence was observed in the age group over 45 years (8.33%) [36]. The results of a meta-analysis study also showed that the prevalence of HPV In Asian countries decreases with age [37]. The results of our study were in line with studies conducted in Iran and Asian countries and showed a decrease in the prevalence of HPV with age. This can be due to less sexual activity or spontaneous recovery of HPV in older people [37]. Additionally, the increasing risk factors of cervical cancer including smoking, use of birth control pills, and sexually transmitted infections (HIV, *Chlamydia trachomatis*, etc.) may play a role in the recent increase in the incidence of cervical cancer in younger age groups and the higher prevalence of cervical cancer [30].

In the present study, the prevalence of HPV has no significant relationship with the history of genital infections, and only 4.76% of women with HPV have mentioned a history of genital infections. According to various studies, the prevalence of genital infections is to be more expected in women with HPV. It seems that the lack of awareness that many participants have regarding different types and symptoms of genital infections and bias recalling will be effective in this finding.

In the present study, the number of deliveries is related to women being infected with HPV, and women with HPV have had fewer deliveries. Hamkar et al. in a study on women with normal cytology showed that the prevalence of HPV is higher in women without a history of childbirth [24]. Other studies did not confirm the results of our study [38]. It seems that cultural and social issues may be influential in this regard and further research is needed.

The high sample size and wide geographical areas are the strengths of our study. One of the limitations is the lack of study of women in some provinces, especially Tehran province.

## 5. Conclusion

In the study, the prevalence of HPV is estimated in the general population of women, aged 15–59 in 11 provinces of Iran to be 4.21%. Also, the prevalence of high-risk and intermediate-risk HPV was shown to be 3.04%, and the prevalence of low-risk HPV was estimated to be 1.17% of the total female population. Hormozgan province has the highest and Isfahan province has the lowest HPV infections. The most prevalence of HPV was related to the age group of 25–34 years, and the lowest prevalence was related to the age group of 55 years and above. Its higher prevalence in young age groups indicates the need to pay attention to planning and policy-making for vaccination and screening programs.

## Figures and Tables

**Table 1:** Characteristics in HPV positive and negative patients.

Characteristics	Overall *N* = 2562	HPV positive *N* = 108 (4.2%)	HPV negative *N* = 2454 (95.8%)	*p* value	OR	CI 95%
Age (mean ± SD)	35.6 ± 10.9	34.5 ± 10.2	35.6 ± 10.9	0.38^*∗*^	—	—
Age group, years						
15–24 25–34 35–44 44–55 ≥55	458 820 673 459 149	22 (4.8) 37 (4.5) 28 (4.2) 13 (2.8) 5 (3.4)	436 (95.2) 783 (95.5) 645 (95.8) 446 (97.2) 144 (96.6)	0.56	—	—
First menstrual age	13	14	13	0.36	—	—
First menstrual age	18	18.5	18	0.87	—	—
Province						
Hormozgan Ardabil Yazd Isfahan East Azerbaijan Semnan Qazvin Khuzestan Kurdistan Kerman Guilan	232 377 226 274 125 231 225 303 205 176 257	22 (9.5) 18 (4.8) 7 (3.1) 6 (2.2) 5 (4.0) 7 (3.0) 7 (3.0) 9 (3.0) 5 (2.4) 8 (4.5) 14 (5.4)	210 (90.5) 359 (95.2) 219 (96.9) 268 (97.8) 120 (96.0) 224 (97.0) 224 (97.0) 294 (97.0) 200 (97.6) 168 (95.5) 243 (94.6)	**0.004**	—	—
Education						
Uneducated Primary Secondary Educated	514 1166 604 279	13 (2.5) 55 (4.7) 23 (3.8) 15 (5.4)	501 (97.5) 1111 (95.3) 581 (96.2) 264 (94.6)	0.13	—	—
Employment status Housekeeper Occupied	2300 260	89 (3.9) 17 (6.5)	2211 (96.1) 244 (93.5)	**0.042**	**1.73**	**1.01–2.96**
Marital status						
Married Widow Divorced	2461 69 18	97 (3.9) 5 (7.2) 2 (11.1)	2364 (96.1) 64 (92.8) 16 (88.9)	0.12	—	—
Abortion history						
Yes No	650 1915	23 (3.5) 83 (4.3)	627 (96.5) 1832 (95.7)	0.38	0.81	0.51–1.29
Stillbirth history						
Yes No	109 2458	5 (4.6) 101 (4.1)	104 (95.4) 2347 (95.9)	0.81	1.12	0.45–2.81
STD history						
Yes No	21 2547	1 (9.1) 105 (4.1)	20 (91.9) 2442 (95.9)	0.59 ¥	—	—
Number of pregnancy	3 ± 2	2 ± 2	3 ± 2	**0.01**	—	—
HPV status						
HPV type						
High risk Intermediate risk Low risk	108	55 (50.9) 23 (21.3) 30 (27.8)	—	—	—	—

^*∗*^Mann–Whitney Test. ¥Fisher's Exact Test. Abbreviation: HPV: human papillomavirus; SD: standard deviation; STD: sexually transmitted diseases.

**Table 2:** Characteristics in all types of HPV positive patients.

Characteristics	LR HPV	IR HPV	HR HPV	*p* value	*p* value
Age					
Count (mean ± SD)	37 (34.5 ± 9.7)	20 (37 ± 10.2)	49 (33.5 ± 10.8)	106 (34.5 ± 10. 3)	0.45
First menstrual age					
Count (mean ± SD)	24 (13.2 ± 1.5)	15 (13.7 ± 1.0)	36 (13.8 ± 1.4)	75 (13.6 ± 1.4)	0.28
Age of marriage					
Count (mean ± SD)	35 (19.9 ± 4.5)	19 (19.7 ± 5.3)	49 (19.7 ± 4.9)	103 (19.8 ± 4.8)	0.97
Number of pregnancies					
Count (mean ± SD)	37 (2.4 ± 2.2)	20 (2.9 ± 2.3)	49 (2.3 ± 1.7)	106 (2.4 ± 2.0)	0.49
Age group count (%)					
15–24 25–34 35–44 45–54 ≥55	8 (36.4) 12 (32.4) 13 (43.3) 3 (25.0) 1 (20.0)	2 (9.1) 7 (18.9) 6 (20.0) 4 (33.3) 1 (20.0)	12 (54.5) 18 (48.6) 11 (36.7) 5 (41.7) 3 (60.0)	22 (100) 37 (100) 30 (100) 12 (100) 5 (100)	0.75
Province count (%)					
Hormozgan Ardabil Yazd Isfahan East Azerbaijan Semnan Qazvin Khuzestan Kurdistan Kerman Guilan	9 (40.9) 5 (27.8) 0 (0.0) 4 (66.7) 1 (20.0) 2 (28.6) 5 (71.4) 4 (44.4) 2 (40.0) 1 (12.5) 4 (28.6)	3 (13.6) 3 (16.7) 2 (28.6) 0 (0.0) 2 (40.0) 1 (14.3) 0 (0.0) 0 (0.0) 2 (40.0) 3 (37.5) 4 (28.6)	10 (45.5) 10 (55.6) 5 (71.4) 2 (33.3) 2 (40.0) 4 (57.1) 2 (28.6) 5 (55.6) 1 (20.0) 4 (50.0) 6 (42.9)	22 (100) 18 (100) 7 (100) 6 (100) 5 (100) 7 (100) 7 (100) 9 (100) 5 (100) 8 (100) 14 (100)	0.33
Education count (%)					
Uneducated Primary Secondary Educated	1 (7.7) 25 (45.5) 6 (26.1) 5 (33.3)	5 (38.5) 8 (14.5) 5 (21.7) 2 (13.3)	7 (53.8) 22 (40.0) 12 (52.2) 8 (53.3)	13 (100) 55 (100) 23 (100) 15 (100)	0.14
Employment status count (%)					
Housekeeper Occupied	30 (33.7) 7 (41.2)	18 (20.2) 2 (11.8)	41 (46.1) 8 (47.1)	89 (100) 17 (100)	0.68
Marital status count (%)					
Married Divorced Widow	31 (32.0) 2 (100) 3 (60.0)	20 (20.6) 0 (0.0) 0 (0.0)	46 (47.4) 0 (0.0) 2 (40.0)	97 (100) 2 (100) 5 (100)	0.19
Abortion history count (%) No Yes	27 (32.5) 10 (43.5)	17 (20.5) 3 (13.0)	39 (47) 10 (43.5)	83 (100) 23 (100)	0.55
Stillbirth history count (%)					
No Yes	35 (34.7) 2 (40.0)	20 (19.8) 0 (0.0)	46 (45.5) 3 (60.0)	101 (100) 5 (100)	0.54
STD history count (%)					
No Yes	1 (100) 36 (43.3)	0 (0.0) 20 (19.0)	0 (0.0) 49 (46.7)	1 (100) 105 (100)	0.39

Abbreviation: HPV: human papillomavirus; LR: low risk; IR: intermediate risk; HR: high risk; SD: standard deviation; STD: sexually transmitted diseases. High risk: 16, 18, 31, 33, 35, 45, 52, 58, 39, 51, 56, 59. Intermediate risk: 68, 26, 30, 34, 53, 66, 67, 69, 70, 73, 82, 85, 97. Low risk: 6, 11, 40, 42, 43, 44, 54, 55, 61, 62, 67, 69, 71, 72, 81, 84, 89.

## Data Availability

The data are available and there is no restriction on additional information.
